# Different structural brain patterns and their association with executive function and general cognitive ability in cognitively normal elderly adults

**DOI:** 10.3389/fnagi.2026.1693197

**Published:** 2026-02-02

**Authors:** Wenjie Pan, Cheng Xu, Cheng Zhu, Jiaojiao Wang

**Affiliations:** 1 Ruian Fifth People’s Hospital, Wenzhou, China; 2 The Affiliated Kangning Hospital of Wenzhou Medical University, Zhejiang Provincial Clinical Research Center for Mental Health, Wenzhou, China

**Keywords:** cognitively normal elderly adults, cortical thickness, general cognitive ability, gray matter volume, structural brain

## Abstract

**Objectives:**

Structural gray matter changes were significantly observed in cognitively normal elderly adults, but the associations between different structural brain patterns, executive function, and general cognitive ability in older adults have not been fully explored.

**Methods:**

A total of 119 cognitively normal elderly adults and 162 healthy younger adults were enrolled in this study and underwent extensive cognitive assessment and structural magnetic resonance imaging. Surface-based morphometry was used to reconstruct the cortical surface and derive structural indices, including cortical thickness (CT), surface area (CSA), and gray matter volume (GMV), which were then used to examine brain region differences between younger and older adults. Moderated mediation model analysis was conducted to explore the associations between different structural brain patterns, executive function, and general cognitive ability.

**Results:**

Compared with younger adults, older adults showed significantly lower CT mainly in frontal regions and lower GMV of bilateral thalamus and bilateral putamen. The analysis of the mediating effect found that the GMV of the right thalamus is significantly associated with executive function, and executive function is also significantly associated with general cognitive ability. Furthermore, for younger adults, executive function mediated the effect of GMV of the right thalamus on general cognitive ability, whereas for older adults, the mediating effect of executive function was not significant.

**Conclusion:**

Cognitively normal elderly adults exhibited different structural brain patterns, which were further associated with executive function and general cognitive ability. These findings shed light on the relationship between brain structural pattern changes and cognition in cognitively normal elderly adults.

## 1 Introduction

Aging is a complex, progressive physiological process involving changes in multiple systems, with cognitive decline being particularly notable (Gonzales et al., 2022; Oschwald et al., 2019). Throughout this process, individuals experience gradual declines in memory, learning ability, attention, and executive function, which directly influence their daily lives and overall wellbeing (Craik and Salthouse, 1992; Iulita et al., 2023). Executive function is an essential cognitive function that refers to a set of top-down mental processes involved in information processing, regulation, and control. These functions form the basis of higher-order cognitive activities, such as planning, reasoning, decision-making, and problem-solving (Botvinick et al., 2001). Recent studies have shown that executive function significantly declines with aging. This decline contributes to general cognitive deterioration during aging (Clark et al., 2012; Salthouse et al., 2003) and plays a crucial role in neurodegenerative diseases, such as Alzheimer’s disease (Chainay and Gaubert, 2020). However, executive function represents only one aspect of the broader concept of general cognitive ability, which also includes attention, memory, language skills, processing speed, and visuospatial abilities (Harada et al., 2013). These components together contribute to an individual’s overall cognitive health and daily functioning. As aging progresses, all of these cognitive domains are affected, and executive function often serves as an early indicator of broader cognitive decline (Clark et al., 2012). Therefore, understanding the neurobiological mechanisms behind the decline in executive function, as well as its relationship with general cognitive ability, is crucial for gaining insights into the aging process and its impact on cognitive health.

In recent years, researchers have recognized that changes in cognitive abilities during aging are not isolated events, but are closely linked to changes in brain structure. As people grow older, brain structural indicators such as cortical thickness (CT) and gray matter volume (GMV) gradually change. These changes may underpin the decline in cognitive function (Cardenas et al., 2011; Tatti et al., 2016). The prefrontal aging theory suggests that degenerative change in the prefrontal cortex is a key factor in the decline of executive function (West, 1996). This theory emphasizes the prefrontal region’s core role in regulating executive function and suggests that structural changes during aging may weaken its regulatory capacity, subsequently affecting cognitive processes such as decision-making, planning, working memory, and inhibitory control. Besides, the dedifferentiation hypothesis suggests that neural differentiation decreases in cognitively normal elderly adults and predicts performance across multiple cognitive domains in an age-invariant manner (Koen and Rugg, 2019). Additionally, the compensation-related utilization of neural circuit’s hypothesis proposes that aging individuals may recruit additional neural resources to compensate for declines in specific brain regions, especially in the context of executive function (Reuter-Lorenz and Cappell, 2008). These alternative frameworks broaden the understanding of how aging impacts brain function and support the notion that the relationship between brain structure and cognitive abilities is complex and multifaceted. Furthermore, numerous studies have found that structural changes in other brain regions are closely related to aging and cognitive decline. For instance, a study of 207 adults aged 23 to 87 years found declines in cortical surface area (CSA), CT, and GMV in various brain regions, including the occipital, parietal, and temporal lobes, as well as the hippocampus, with increasing age. Although the rate of decline varied across different brain regions, the overall trend was consistent (Storsve et al., 2014). Another study comparing brain structures in middle-aged and elderly groups found that GMV of the elderly group was significantly lower than that of the middle-aged group, particularly in regions associated with language processing and executive function (Ramanoël et al., 2018). A longitudinal study of cognitively normal elderly individuals found widespread atrophy in regions such as the temporal lobes, hippocampus, thalamus, and cerebellum. While memory, spatial, and language abilities remained stable, executive function declined significantly over a 4-year period (Neufeld et al., 2022).

Despite significant progress in investigating brain structural changes during aging, existing research still has several notable limitations. First, many studies have focused solely on the relationship between brain structural changes and cognitive function in older adults, without including a comparison group of younger adults (Leong et al., 2017; Schulz et al., 2022). This makes it difficult to understand how the relationship between brain structural changes and cognitive function differs across age groups. Furthermore, while numerous studies have identified significant correlations between changes in brain gray matter structure and declines in overall cognition and executive function, the precise interactions between these variables, as well as the potential moderating effect of age, remain unclear. Additionally, current research predominantly focuses on changes in one specific structural indicator. However, different structural indicators often exhibit different developmental trajectories due to their genetic independence (Chen et al., 2015; Storsve et al., 2014). Lifespan studies using multimodal approaches have also shown that combining multiple structural indicators can reveal the interactions between different control processes. In contrast, single structural indicators may fail to fully capture cognitive changes during the aging process (Hsieh and Lin, 2017; Hsieh et al., 2023; Yao and Hsieh, 2022; Yao et al., 2020). Therefore, it is necessary to further explore how different structural indicators impact cognitive and executive function during aging.

Hence, this study aimed to investigate unique brain structural patterns in cognitively normal elderly adults and explore how these changes are associated with executive function and general cognitive ability. By comparing different structural brain patterns of cognitively normal elderly and younger adults and constructing a moderated mediation model, we hypothesized that (1) compared with younger adults, cognitively normal elderly adults exhibited different structural brain patterns in widespread brain regions; and (2) the potential moderating effect of aging was observed on the relationship between executive function and general cognitive ability.

## 2 Methods

### 2.1 Participants

For this study, we utilized data obtained from the OpenNEURO database.^1^ Our analysis included 119 cognitively normal elderly adults (Mean age = 68.66 years old, Min age = 60 years old, Max age = 89 years old, SD = 6.46; 54.62% female) and 162 healthy younger adults (Mean age = 22.64 years, Min age = 18 years old, Max age = 34 years old, SD = 3.35; 56.79% female). Participants were screened to rule out individuals with a history of neurological or other medical illness known to impact cognition, acute or chronic psychiatric illness, those undergoing current or recent treatment with psychotropic medication, and those having recently experienced significant changes to health status at the time of the eligibility interview. Both age groups were screened for depressive symptoms using the Beck Depression Inventory and the Geriatric Depression Scale, respectively. Furthermore, all participants were additionally administered the Mini-Mental State Examination (MMSE). Finally, all participants had no symptoms of depression or cognitive impairment and were right-handed with normal or corrected-to-normal vision. Participants who exhibited excessive head motion during scanning were excluded from the analysis. Procedures were administered in compliance with the Institutional Review Board at Cornell University and the Research Ethics Board at York University, including written informed consent obtained from each participant. Detailed information regarding the informed consent process with all participants can be found in previous research conducted in this area (Spreng et al., 2022).

### 2.2 Cognitive assessment

All participants enrolled in this study underwent an extensive cognitive assessment over 3 to 4 days before brain scanning, and passed quality assessment (Spreng et al., 2022). Lab assessments included the NIH Toolboxes of Cognition and Emotion and auxiliary measures (Gershon et al., 2013). The NIH Cognition Toolbox included the Rey Auditory Verbal Learning and Picture Sequence Memory, Flanker Inhibitory Control and Attention, Dimensional Change Card Sort, List Sort Working Memory, Picture Vocabulary, and Oral Reading Recognition tests. Composite scores of fluid and crystallized intelligence were also tabulated within the toolbox. General cognitive ability represents the sum of crystallized cognition and fluid cognition. The NIH Emotion Toolbox included surveys of Positive Affect, General Life Satisfaction, Meaning and Purpose, Emotional Support, Instrumental Support, Friendship, Perceived Rejection, Perceived Hostility, Perceived Stress, Self-Efficacy, Anger-Affect, Fear-Somatic Arousal, and Fear-Affect. Additionally, participants completed Verbal Paired Associates from the Wechsler Memory Scale-IV (Wechsler, 1955), the Associative Recall Paradigm (Brainerd et al., 2014), Shipley-Vocabulary (Shipley et al., 2009), Trail Making Test B-A (Reitan, 1958), the Reading Span Task (Daneman and Carpenter, 1980), and the Symbol Digit Modalities Test (Smith, 1982). In our study, episodic memory included scores on Verbal Paired Associates, Associative Recall, NIH Cognition Rey Auditory Verbal Learning, and NIH Cognition Picture Sequence Memory; Semantic memory included scores on Shipley Vocabulary, NIH Cognition Picture Vocabulary, and NIH Cognition Oral Reading Recognition; Executive function included scores on the Trail Making Task, NIH Cognition Flanker Inhibitory Control and Attention, NIH Cognition Dimensional Change Card Sort, and NIH Cognition List Sort Working Memory. Participants were additionally administered the MMSE (Folstein et al., 1975). Participants with MMSE scores below 27/30 were excluded if fluid cognition scores also fell below an age-adjusted national percentile of 25%.

### 2.3 MRI data acquisition

T1 anatomical scans were acquired using a T1-weighted volumetric magnetization-prepared rapid gradient echo sequence. For the GE scanner, the parameters were as follows: repetition time (TR) = 2,530 ms, echo time (TE) = 3.4 ms, flip angle = 7°, voxel size = 1 mm isotropic, 176 slices, and a scanning time of 5 min and 25 s. The scan was performed with 2x acceleration and sensitivity encoding. For the Siemens scanner, anatomical scans were acquired using a similar T1-weighted volumetric magnetization-prepared rapid gradient echo sequence with TR = 1900 ms, TE = 2.52 ms, flip angle = 9°, voxel size = 1 mm isotropic, 192 slices, and a scanning time of 4 min and 26 s. This scan was performed with 2x acceleration and generalized auto-calibrating partially parallel acquisition encoding, using an integrated parallel acquisition technique acceleration factor of 2.

### 2.4 MRI data preprocessing and measurement of CSA, CT, and GMV

Each participant’s structural Magnetic Resonance Imaging (sMRI) data were preprocessed using FreeSurfer v7.2.0 software package.^2^ Detailed information regarding the surface-based morphology analysis can be found in previous studies where the specifics of this analysis were documented (Dale et al., 1999; Fischl and Dale, 2000; Fischl et al., 1999). The FreeSurfer pipeline processing involved several steps, including removal of non-brain tissue, Talairach transformation, intensity normalization, gray/white matter boundary tessellation, topology correction, surface deformation, registration to a common spherical atlas, and cortical surface reconstruction. To obtain measurements of CT and CSA, the cortical morphologies were smoothed using a 10-mm full-width-at-half-maximum Gaussian kernel, following methodologies described in previous research (Glasser et al., 2013; Li et al., 2020; Zhang et al., 2021). CT was calculated at each vertex in the cortex by measuring the distance between the pial surface and the gray–white matter surface. This approach provides a local assessment of CT across the entire cortical surface. CSA was estimated by averaging the area of all faces connected to a specific vertex on the white matter surface. Additionally, GMV for subcortical regions was calculated by the Automated Segmentation of subCortical Structures atlas, which was performed by the FreeSurfer pipeline automatically (Fischl et al., 2002). All outputs underwent meticulous inspection throughout the preprocessing phase, and manual corrections were applied as necessary. Subsequently, the average values of CT and CSA within 34 cortical parcellations were determined in each hemisphere and defined by the Desikan atlas (Desikan et al., 2006). All sMRI indexes, including CT, CSA, and GMV, were exported for subsequent analysis.

### 2.5 Statistical analysis

Demographic and cognitive characteristics of all participants were analyzed using R (Version 4.1.3; R Core Team, 2022) and RStudio (“Ghost Orchid” Release; RStudio Team, 2021). For demographic and cognitive characteristics, an independent two-sample T-test was performed, and a threshold of *p* < 0.05 was used to indicate significance after Bonferroni correction. Then, any of the above variables that showed significant group differences were included as covariates in the following analysis of sMRI variables.

For sMRI variables, a linear mixed effects (LME) model was conducted for each sMRI variable. The above independent two-sample T-test has found that education level, crystallized cognition, semantic memory, fluid cognition, general cognitive ability, executive function, and episodic memory showed significant group differences. Hence, these characteristics were included as covariates in the LME model. A threshold of *p* < 0.05 was used to indicate significance after Bonferroni correction.

Then, correlation analysis and moderated mediation model analysis were performed using the Mediation package in R (version 4.1.3). In this study, a moderated mediation model was constructed using the mediation package in R, and the mediating effects of executive function and the moderating role of age group were tested. We performed parameter estimation using the bootstrap method with 5,000 replicate samples and a 95% confidence interval (CI). We considered the effect to be significant if the CI did not include 0 and not significant if the CI included 0.

## 3 Results

### 3.1 Participants and characteristics

The demographic information and cognitive assessments of all participants are depicted in Table 1. There were no significant group differences observed in gender (*χ*^2^_(1)_ = 0.06, *p* = 0.81) and total score of MMSE (*t* = −3.01, *p* = 0.23) between older adults and younger adults. Older adults showed a higher level of education (*t* = 6.84, p_adj_ < 0.001), crystallized cognition (*t* = 7.64, p_adj_ < 0.001), and semantic memory (*t* = 9.45, p_adj_ < 0.001) than younger adults, while older adults exhibited lower fluid cognition (*t* = −14.82, p_adj_ < 0.001), general cognitive ability (*t* = −6.38, padj < 0.001), executive function (*t* = −14.86, p_adj_ < 0.001), and episodic memory (*t* = -16.82, p_adj_ < 0.001). There were significant group differences in other cognitive assessment variables between older and younger adults (p_adj_ < 0.05; Supplementary Table S1).

**Table 1:** Demographic and cognitive assessments in older and younger adults.

Demographics and cognition	Older adults	Younger adults	*t*/*χ*^2^	p_adj_
Age	68.66 (6.46)	22.64 (3.35)	71.02	0.001
Gender (F/M)	65/54	92/70	0.06	0.810
Education	17.28 (2.87)	15.20 (1.92)	6.84	0.001
MMSE	28.66 (1.23)	29.10 (1.21)	−3.01	0.230
Crystallized cognition	135.64 (10.36)	126.15 (9.88)	7.64	0.001
Fluid cognition	96.57 (10.19)	118.39 (14.03)	−14.82	0.001
General cognitive ability	121.19 (11.54)	131.11 (14.03)	−6.38	0.001
Episodic memory	−0.71 (0.66)	0.53 (0.53)	−16.82	0.001
Semantic memory	0.49 (0.70)	−0.34 (0.77)	9.45	0.001
Executive function	−0.51 (0.47)	0.39 (0.54)	−14.86	0.001

F, female; M, male; MMSE, Mini-Mental State Exam total score. Episodic memory represented a composite score of Verbal Paired Associates, Associative Recall, NIH Cognition Auditory Verbal Learning, and NIH Cognition Picture Sequence Memory variables. Semantic memory represented a composite score of Shipley Vocabulary, NIH Cognition Picture Vocabulary, and NIH Cognition Oral Reading Recognition variables. Executive function represented a composite score of the Trail Making Task, NIH Cognition Flanker, NIH Cognition Dimensional Change Card Sort, and NIH Cognition List Sort Working Memory variables. General cognitive ability represents the sum of crystallized cognition and fluid cognition.

### 3.2 Different structural brain patterns in cognitively normal elderly adults

**Figure 1:**
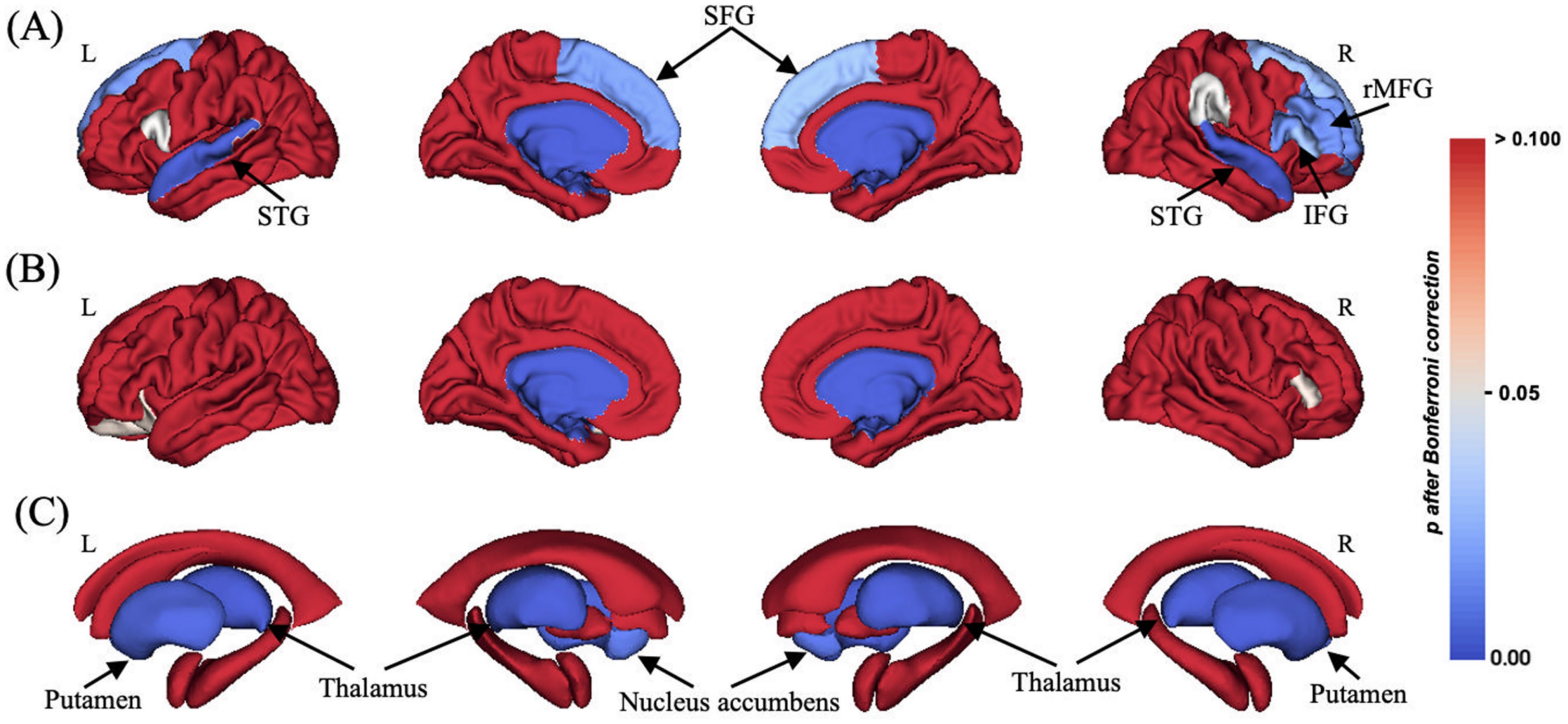
Structural brain differences in **(A)** cortical thickness, **(B)** cortical surface area, and **(C)** subcortical gray matter volume between groups (older and younger adults). The color bar of brain regions represented *p*-value after Bonferroni correction. L, left; R, right; SFG, superior frontal gyrus; STG, superior temporal gyrus; IFG, inferior frontal gyrus; rMFG, rostral middle frontal gyrus.

In terms of CT, older adults showed significantly lower CT of bilateral superior frontal gyrus (SFG), bilateral superior temporal gyrus (STG), right opercular and triangular part of inferior frontal gyrus (IFG), and right rostral middle frontal gyrus (rMFG) compared with younger adults (all *p* < 0.05 after Bonferroni correction, Table 2 and Figure 1A). There were no significant group differences observed in terms of CSA (Figure 1B).

**Table 2:** LME analysis of group difference of structural gray matter in older and younger adults.

Structural brain regions	Older adults	Younger adults	*t*	p_adj_
Cortical thickness				
Left SFG	2.60 (0.14)	2.79 (0.12)	3.98	0.020
Left STG	2.62 (0.13)	2.78 (0.15)	4.67	0.001
Right opercular part of IFG	2.50 (0.15)	2.65 (0.12)	4.02	0.010
Right triangular part of IFG	2.35 (0.16)	2.51 (0.12)	3.84	0.030
Right rMFG	2.28 (0.12)	2.38 (0.09)	4.04	0.010
Right SFG	2.58 (0.15)	2.77 (0.12)	3.85	0.030
Right STG	2.66 (0.14)	2.85 (0.14)	5.46	0.001
Cortical surface area				
No significant group differences were observed				
Subcortical gray matter volume				
Left thalamus	7070.37 (883.98)	8172.44 (785.99)	5.27	0.001
Left putamen	4319.68 (605.49)	5132.17 (666.65)	4.46	0.001
Left nucleus accumbens	406.11 (146.31)	561.22 (155.52)	4.18	0.010
Right thalamus	6926.69 (805.22)	7795.00 (731.05)	4.76	0.001
Right putamen	4371.69 (639.28)	5207.18 (577.24)	5.07	0.001
Right nucleus accumbens	464.09 (104.65)	574.37 (110.38)	4.26	0.010

SFG, superior frontal gyrus; STG, superior temporal gyrus; IFG, inferior frontal gyrus; rMFG, rostral middle frontal gyrus.

In terms of subcortical GMV, older adults exhibited significantly lower GMV of bilateral thalamus, bilateral putamen, and bilateral nucleus accumbens compared with younger adults (all *p* < 0.05 after Bonferroni correction, Table 2 and Figure 1C).

### 3.3 Different structural brain patterns association with executive function and general cognitive ability

**Figure 2:**
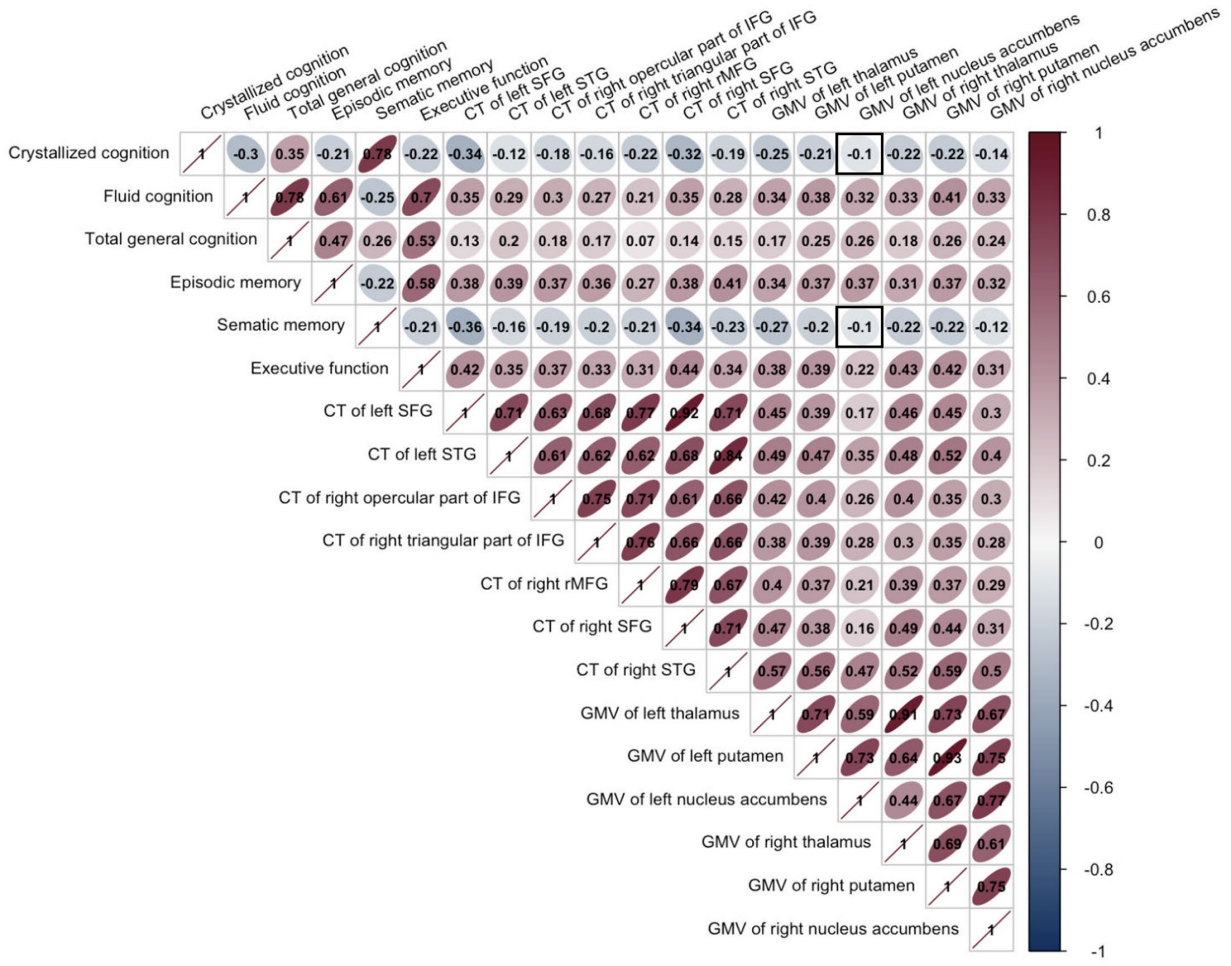
Person correlation results of all variables showed significant group differences after Bonferroni correction. It showed that only the GMV of the left nucleus accumbens was not significantly correlated with crystallized cognition and semantic memory, which has been indicated by a black square. The color bar represented the r-value of the correlation results. CT, cortical thickness; GMV, gray matter volume; SFG, superior frontal gyrus; STG, superior temporal gyrus; IFG, inferior frontal gyrus; rMFG, rostral middle frontal gyrus.

A different structural brain pattern was significantly associated with cognitive assessment variables after Bonferroni correction (p_adj_ < 0.05; Figure 2).

**Figure 3:**
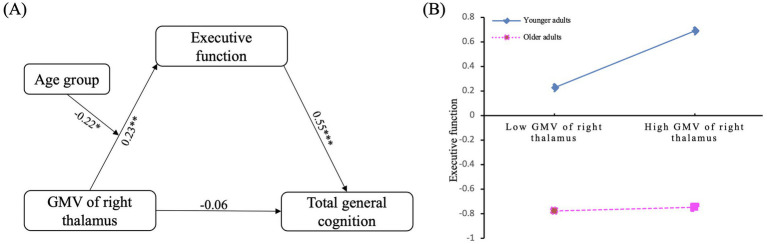
The moderated mediation model **(A)** and the moderating effect of age group **(B)**. GMV, gray matter volume (participants were grouped into high and low categories based on one standard deviation above and below the mean).

To investigate the relationships between different structural brain patterns, executive function, and general cognitive ability, moderated mediation models were used, implemented through the Mediation package in R. The results found (see Table 3; Figure 3A) that GMV of the right thalamus cannot predict general cognitive ability (*β* = 0.50, *p* = 0.307). While the GMV of the right thalamus can significantly predict executive function (*β* = 0.23, *p* = 0.002), and executive function can also significantly predict general cognitive ability (*β* = 0.55, *p* < 0.001). In addition, the interaction of GMV of the right thalamus and age group can also significantly predict executive function (*β* = −0.22, *p* = 0.044). Furthermore, the magnitude of the mediation pathway effects at different levels of the age group was illustrated in Table 4. For younger adults, executive function mediated the effect of GMV of the right thalamus on general cognitive ability, whereas for older adults, the mediating effect of executive function was not significant.

**Table 3:** Moderated mediation model.

Regression equation		Overall fit indices			Significance of the regression coefficients	
Outcome variables	Predictors	*R*	*R^2^*	*F*	*β*	*t*
Executive function	GMV of the right thalamus	0.67	0.45	71.70^***^	0.23	3.10^**^
	Age group				−1.22	−11.26^***^
	(GMV of right thalamus) × (Age group)				−0.22	−2.03^*^
General cognitive ability	GMV of right thalamus	0.53	0.28	51.94^***^	−0.06	−1.02
	Executive function				0.55	9.61^***^

*p < 0.05; **p < 0.01; ***p < 0.001.

**Table 4:** Mediating effect analysis of the age group.

Age group	Mediation variable	Effect	SE	95%CI
Younger adults	Mediating effect of executive function	0.13	0.05	[0.043, 0.218]
Older adults	Mediating effect of executive function	0.01	0.04	[−0.075, 0.072]
	Direct effect of the model	−0.06	0.06	[−0.170, 0.054]

SE, standard error; CI, confidence interval.

To further test the moderating effect of age group, a simple slope analysis was conducted. As shown in Figure 3B, for young individuals, GMV of the right thalamus can significantly predict executive function (simple slope = 0.23, *p* = 0.002). While for old individuals, the GMV of the right thalamus could not significantly predict executive function (simple slope = 0.01, *p* = 0.847).

## 4 Discussion

This study analyzed brain structural differences between cognitively normal elderly adults and younger adults, revealing unique brain structural patterns in cognitively normal elderly adults in terms of CT, CSA, and GMV. The results show that, in terms of CT, cognitively normal elderly adults exhibited significantly lower CT in brain regions, such as the prefrontal cortex and temporal lobe. Moreover, compared to younger adults, CT of the rMFG in older adults was significantly lower, while there were no significant group differences in CSA. Regarding subcortical GMV, older adults showed lower GMV in the bilateral thalamus, bilateral putamen, and bilateral nucleus accumbens compared with younger adults. Furthermore, the moderated mediation analysis revealed an important and novel finding: in younger adults, executive function mediated the relationship between right thalamus GMV and general cognitive ability. However, in older adults, this mediating effect was not significant, suggesting that aging may disrupt the normal relationship between brain structure and cognitive function. This finding provides important insights into how aging affects cognitive processes, highlighting the need for further exploration of compensatory mechanisms in older adults. These results not only demonstrate different brain structural patterns in cognitively normal elderly adults but also offer a novel perspective on the interactions between brain structural changes, executive function, and general cognitive ability during aging. By revealing the differential mediation effects in younger versus older adults, this study contributes a new understanding of the neurobiological mechanisms underlying cognitive aging.

In our study, we observed that crystallized intelligence and semantic memory were notably more stable across the aging process compared to other cognitive domains, such as fluid intelligence and executive function. Specifically, older adults performed similarly to younger adults in tasks measuring these abilities, which aligns with previous research suggesting that crystallized intelligence, which is based on accumulated knowledge and experience, tends to remain stable or even improve with age (Harada et al., 2013; Oschwald et al., 2019). This stability is often attributed to the continuous accumulation of knowledge throughout one’s life, which may compensate for some of the declines observed in other cognitive functions. Similarly, semantic memory, which refers to the ability to recall general knowledge and facts, also remains relatively unaffected by aging, likely due to the robustness of long-term memory systems that store well-established information (Harada et al., 2013; Rönnlund et al., 2005). Thus, while fluid intelligence and executive function decline with age, crystallized intelligence and semantic memory appear to be preserved, reflecting a complex interaction between different cognitive processes that may contribute to maintaining cognitive function in older adults.

The study also found that cognitively normal elderly adults had significantly lower CT of the bilateral SFG, bilateral STG, right opercular and triangular part of the IFG, compared with controls. The SFG connects to the supplementary motor area, which is involved in motor task activation, and is also associated with working memory and attention-related functions (Corbetta et al., 2008; Li et al., 2013). Reduced CT of SFG may directly affect these functions, leading to lower efficiency in cognitively normal elderly adults when focusing attention or maintaining information. Additionally, the rMFG is considered a core region for contextual memory. Previous studies have found that activation of rMFG changes significantly during memory retrieval, particularly when retrieving information about items, spatial context, and temporal context. Therefore, reduced CT of rMFG may make it more difficult for cognitively normal elderly adults to perform memory retrieval tasks, particularly context recall and complex information processing. This can lead to declines in reasoning, cognitive flexibility, and problem-solving abilities, which are closely related to executive function (Harada et al., 2013). Additionally, the MFG is also considered to play an important role in arithmetic and reading and writing tasks (Koyama et al., 2017; Luo et al., 2020). Therefore, lower CT of bilateral MFG may not only cause older adults to have greater difficulty with memory retrieval tasks, especially episodic recall and complex information processing, but may also lead to declines in reasoning, cognitive flexibility, and problem-solving abilities, all of which are closely related to an individual’s executive function (Harada et al., 2013). Previous research has indicated that the integrity of the IFG not only influences an individual’s inhibitory control and language fluency (Costafreda et al., 2006; Swick et al., 2008; Xu et al., 2018) but also collaborates with the STG in motor planning, speech production, and executive processing tasks related to acquiring speech representations (Hickok, 2009). Furthermore, the STG is associated with attention and information processing speed (Achiron et al., 2013). Therefore, the degeneration of the IFG and STG may lead to impaired performance in language tasks and auditory processing in older adults, thereby affecting cognitive task performance.

This study also found that cognitively normal elderly adults exhibited significantly lower GMV of the bilateral thalamus, putamen, and nucleus accumbens compared to younger adults. The thalamus is a vital relay nucleus and connects the cerebral cortex. Previous studies have also shown significant thalamic atrophy with aging (Choi et al., 2022). Lower thalamic GMV may decrease the efficiency of information processing and affect older adults’ performance in executive tasks, such as task switching, decision-making, and conflict monitoring. Furthermore, the putamen, a major component of the basal ganglia, primarily regulates motor planning and execution, supporting learning processes during various cognitive and emotional challenges (Schultz, 2016; Viñas-Guasch and Wu, 2017). Previous studies have found that neuronal loss and changes in neurotransmission lead to continuous GMV reduction in subcortical nuclei with aging (Cherubini et al., 2009). Putamen degeneration may result in dual declines in motor function and cognitive ability. The nucleus accumbens, a core structure of the brain’s reward system, is responsible for motivation, reward processing, and emotional regulation (Floresco, 2015). Its atrophy may affect motor function, emotional regulation, and the capacity to process rewards. This diminished capacity plays a key role in the decline of response inhibition and related cognitive functions in cognitively normal elderly adults.

The mediation analysis found that, although the GMV of the right thalamus is not directly associated with overall cognitive function, executive function acts as a mediator in the relationship between GMV of the right thalamus and cognitive function. As a core relay station of the brain, the thalamus is highly interconnected with various structures in the central nervous system. The thalamus receives excitatory projections from both the superficial and deep superior colliculus, integrates inputs from the cerebellum and basal ganglia, and projects to the cerebral cortex (Shine et al., 2023). These complex connectivity patterns are crucial not only for sensory processing but also for executive function. The thalamus plays an important role in regulating goal-directed behavior, cognitive flexibility, and task switching. A large body of research has confirmed that the thalamus is involved in memory, information processing speed, attention, and inhibitory control (Philp et al., 2014; Shine et al., 2023). Additionally, the inhibitory control deficit hypothesis suggests that older adults experience a decline in inhibitory control when blocking irrelevant information, removing unnecessary information, and maintaining dominant responses. This decline may lead to reductions in working memory, learning, and comprehension abilities (Hasher and Zacks, 1988). The thalamus plays a central role in inhibiting irrelevant information and selectively incorporating relevant information into working memory (Chatham et al., 2014). Thus, the thalamus likely impacts overall cognitive function indirectly, through the regulation of various aspects of executive functions, particularly cognitive flexibility, goal-directed behavior, and information filtering. These functions indirectly shape overall cognitive performance. This mediating effect underscores the thalamus’s pivotal role in executive control and inhibiting irrelevant information, which contribute to cognitive functions.

Additionally, in this study, for younger adults, the GMV of the right thalamus is significantly associated with executive function. However, this path was not significant for cognitively normal elderly adults. One possible explanation for this result is that older individuals may have small lesions in the thalamus due to the effects of aging, which could affect the relationship between thalamic volume and executive function. Additionally, compensatory changes brought about by neuroplasticity mechanisms could influence the predictive effect of the thalamus on executive function. Recent research has shown that the thalamic nuclei are among the most significantly atrophied nuclei in the brain during aging (Choi et al., 2022), and older adults’ brains may rely on compensatory mechanisms to make up for this structural change (Goldstone et al., 2018). For example, research has found that, in patients with Alzheimer’s disease, functional connectivity is enhanced between the bilateral thalamus and brain regions such as the anterior cingulate gyrus, middle temporal gyrus, inferior temporal gyrus, superior parietal lobule, posterior cingulate, and precuneus (Zhou et al., 2013). Thus, while the thalamus still plays a role in executive function, its predictive relationship with executive function may diminish. Furthermore, stronger thalamic activity does not always correspond to better cognitive performance as age increases. Studies have shown that elderly individuals exhibit stronger thalamus-hippocampus functional connectivity (Goldstone et al., 2018), but this increased activity may indicate more interference from irrelevant information, which contradicts better cognitive performance. Besides, the absence of mediation may reflect compensatory mechanisms or non-linear reorganization in the brain. As aging progresses, the brain undergoes structural and functional reorganization that may engage alternative pathways to maintain cognitive function. Recent studies have shown that age-related shifts in prefrontal–thalamic engagement occur in both structural and functional contexts, suggesting that these shifts may reflect neural reorganization rather than degeneration (Hsieh and Lin, 2017; Hsieh and Yang, 2021). Therefore, the relationship between the thalamic structure and cognitive function in older adults may be more complex than in younger individuals. It is important to note that recent research has highlighted cognitive reserve as a key factor influencing cognitive performance during aging. Cognitive reserve can help mitigate the effects of age-related brain changes, such as reduced thalamic volume. For example, a recent study found that individuals with higher cognitive reserve were able to maintain similar cognitive performance even with smaller thalamic volumes compared to those with larger volumes (Thanissery et al., 2025). This suggests that cognitive reserve may partially compensate for the structural decline in regions such as the thalamus, which could reduce the predictive power of brain volume on executive function, especially in older adults. Therefore, while we controlled for education level, which serves as a proxy for cognitive reserve, future studies should explore how cognitive reserve influences the relationship between brain structure and cognitive performance in aging populations.

However, several limitations should be considered in this study. First, the cross-sectional design of this study limits the ability to draw causal inferences about the relationship between brain structural patterns and cognitive ability during aging. Longitudinal studies would provide a better understanding of how changes in brain structure correlate with cognitive decline in older adults over time. Second, while our sample is representative of cognitively normal elderly adults, the sample size for each group is relatively small compared to many large-scale aging studies. This could limit the reliability and generalizability of our conclusions. Furthermore, some common age-related conditions, such as hypertension or diabetes, which are known to significantly affect brain structure and cognition, were not specifically accounted for in this study. This may raise concerns about the robustness of the findings, as these conditions could potentially confound the observed relationships. Future research should consider including individuals with clinical conditions such as mild cognitive impairment or neurodegenerative diseases, and examine the role of structural changes in both healthy aging and cognitive decline associated with these conditions. Moreover, the current study primarily assumes a linear relationship between brain structure and cognitive function, while older adults may activate alternative frontal pathways or other compensatory mechanisms to counteract structural decline. These complex non-linear trajectories could impact cognitive performance and should be further explored. Future studies should examine these compensatory mechanisms and non-linear trajectories to gain a more comprehensive understanding of the relationship between brain structure and cognitive function during aging (Wang et al., 2024; Yao et al., 2021).

## 5 Conclusion

In conclusion, this study highlighted the different brain structural patterns and their association with executive function and general cognitive ability in cognitively normal elderly adults. Our findings suggested that structural changes, particularly in the thalamus, prefrontal regions, and putamen, contribute to cognitive decline in aging. Notably, executive function played a significant mediating role in this relationship in younger adults, while the same mediating effect was not observed in older adults. These results not only enhanced our understanding of the neurobiological processes involved in cognitive aging but also provided valuable insights into potential strategies for preserving cognitive health in older adults.

## Data Availability

The raw data supporting the conclusions of this article will be made available by the authors, without undue reservation.

## Footnotes

1
https://openneuro.org/datasets/ds003592/versions/1.0.9

2
http://surfer.nmr.mgh.harvard.edu
