# Supplementary material for: Different structural brain patterns and their association with executive function and general cognitive ability in cognitively normal elderly adults
Source: Front Aging Neurosci. 2026 Feb 2;18:1693197. doi: 10.3389/fnagi.2026.1693197 (PMC12907821; doi:10.3389/fnagi.2026.1693197)

Supplemental materials

| **Table S1**. Other variables of demographic and cognitive assessments in older and younger adults | | | | |
| --- | --- | --- | --- | --- |
|  | Older adults | Younger adults | t | p after bonferroni |
| Total correct of Verbal Paired Associates tests | 30.50(8.91) | 43.20(8.13) | -12.25 | <0.001 |
| Delayed total correct of Verbal Paired Associates tests | 9.26(2.70) | 12.98(1.53) | -13.54 | <0.001 |
| Correct Verbal Paired Associates words recalled after a 20-minute delay | 15.43(4.54) | 21.16(3.74) | -11.24 | <0.001 |
| Total number of correctly recalled word pairs | 110.35(51.11) | 178.46(42.26) | -11.77 | <0.001 |
| Immediate recall unadjusted score | 23.98(5.13) | 31.99(5.10) | -12.82 | <0.001 |
| Total number of correct synonyms identified out of 40 questions | 36.15(2.59) | 32.99(3.77) | 8.30 | <0.001 |
| Oral Reading Recognition unadjusted score | 132.87(10.80) | 127.46(10.16) | 4.20 | 0.003 |
| Picture Vocabulary unadjusted score | 134.34(10.14) | 120.75(10.00) | 11.04 | <0.001 |
| Picture Sequence Memory unadjusted score | 98.45(11.26) | 118.81(13.87) | -13.36 | <0.001 |
| List Sorting Working Memory Task unadjusted score | 106.65(9.14) | 117.98(11.35) | -9.15 | <0.001 |
| Dimensional Card Change Sort Task unadjusted score | 99.78(8.23) | 114.59(10.34) | -13.21 | <0.001 |
| Flanker Inhibitory Control Task unadjusted score | 94.15(6.25) | 106.71(13.95) | -10.08 | <0.001 |
| Total number of correctly decoded symbols for Symbol Digits Modality writing portion | 57.01(7.92) | 76.15(12.06) | -16.01 | <0.001 |
| Agreeableness trait | 4.10(0.43) | 3.82(0.56) | 4.47 | <0.001 |
| Neuroticism trait | 2.23(0.60) | 2.69(0.68) | -5.72 | <0.001 |
| Withdrawal facet | 2.15(0.67) | 2.71(0.74) | -6.31 | <0.001 |
| Volatility facet | 2.30(0.70) | 2.67(0.82) | -3.87 | 0.011 |
| Politeness facet | 4.08(0.47) | 3.69(0.62) | 5.71 | <0.001 |
| Industriousness facet | 3.81(0.69) | 3.47(0.65) | 4.00 | 0.007 |
| Drive | 10.13(2.41) | 11.48(2.47) | -4.43 | 0.001 |
| Fun Seeking | 11.07(2.16) | 12.31(2.38) | -4.41 | 0.001 |
| Reward Responsiveness | 16.50(1.93) | 17.64(2.16) | -4.50 | <0.001 |
| Inhibition | 19.65(3.50) | 21.61(3.81) | -4.35 | 0.0016 |
| Fantasy | 2.23(0.82) | 2.67(0.68) | -4.56 | <0.001 |
| Personal Distress | 1.09(0.56) | 1.56(0.63) | -6.29 | <0.001 |
| Tscore of Anger-Affect | 45.14(7.51) | 49.04(7.86) | -4.17 | 0.003 |
| Tscore of Anger Hostility | 44.45(8.19) | 55.16(7.52) | -11.10 | <0.001 |
| Tscore of Physical Aggression | 47.64(6.93) | 55.46(9.18) | -8.05 | <0.001 |
| Tscore of Fear-Affect | 50.44(7.47) | 55.94(7.84) | -6.25 | <0.001 |
| Tscore of Fear-Somatic Arousal | 46.67(5.96) | 55.20(7.79) | -8.94 | <0.001 |
| Tscore of Loneliness | 50.25(7.65) | 54.68(8.36) | -4.56 | <0.001 |
| Tscore of Perceived Stress | 43.64(9.05) | 52.75(9.28) | -8.16 | <0.001 |
| Tscore of Sadness | 44.93(7.25) | 49.53(7.60) | -5.10 | <0.001 |
| Internal density scores | 0.07(0.02) | 0.09(0.02) | -9.58 | <0.001 |
| External density scores | 0.04(0.01) | 0.02(0.01) | 8.96 | <0.001 |
| Internal event density scores | 0.04(0.01) | 0.05(0.02) | -9.44 | <0.001 |
| External event density scores | 0.01(0.01) | 0.01(0.00) | 4.33 | 0.002 |
| External place density scores | 0.00(0.00) | 0.00(0.00) | 4.82 | <0.001 |
| Internal time density scores | 0.00(0.00) | 0.01(0.01) | -4.70 | <0.001 |
| Internal perceptual density scores | 0.01(0.01) | 0.02(0.01) | -4.51 | <0.001 |
| External semantic density scores | 0.01(0.01) | 0.01(0.00) | 8.06 | <0.001 |
| External other density scores | 0.01(0.00) | 0.00(0.00) | 4.43 | 0.001 |

**Figure S1.** The non-significant moderated mediation model of other variables


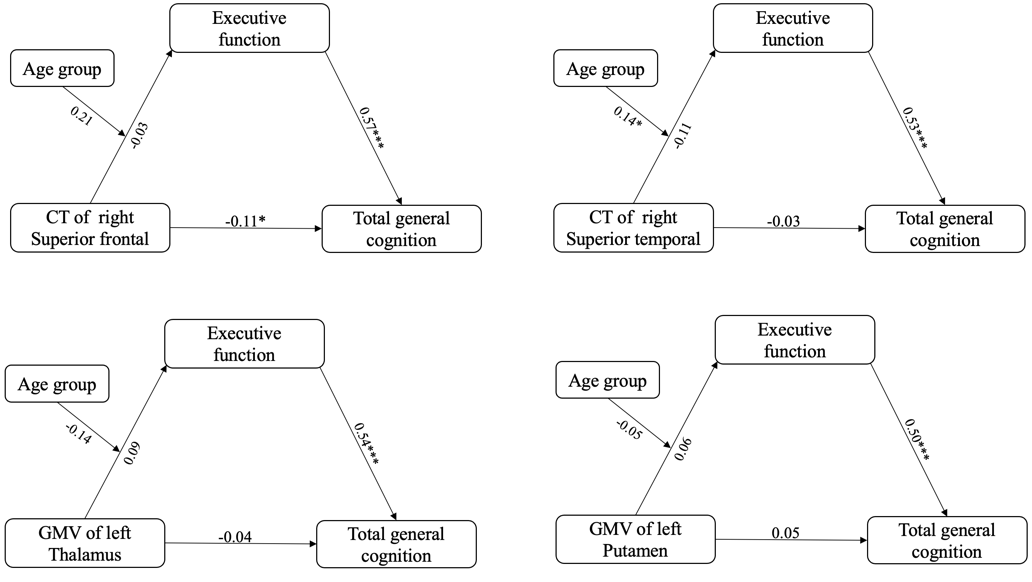


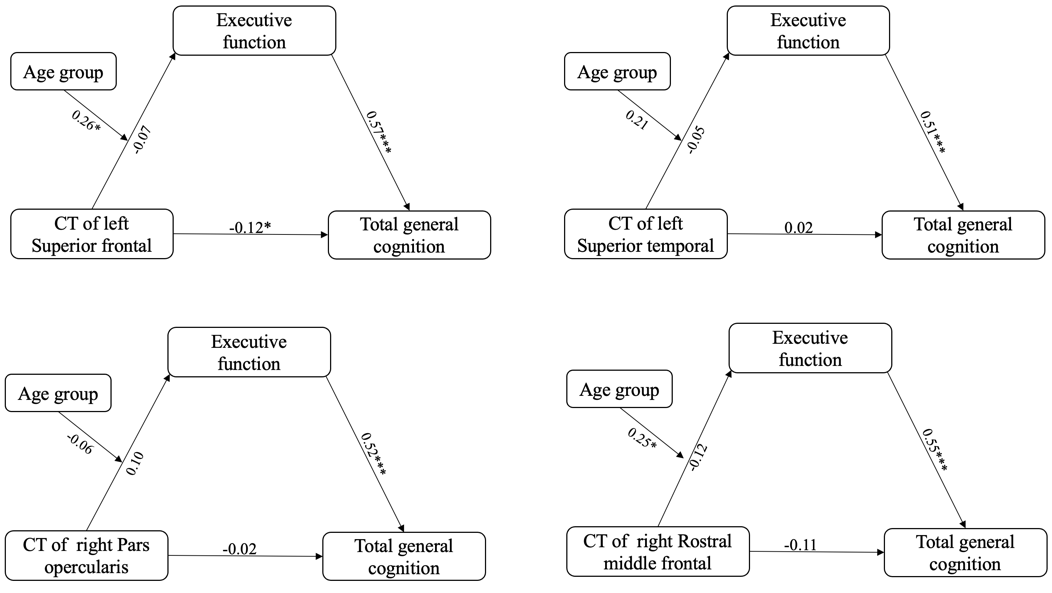


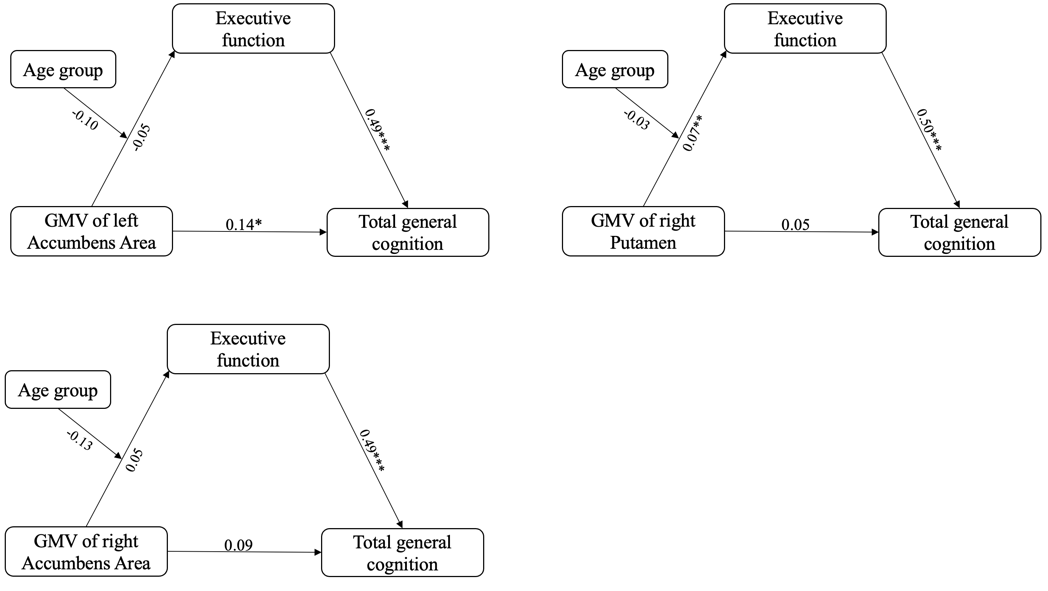

Supplement: Supplementary file 1 [file Table_1.DOCX]
